# Intergenerational Transmission of Obesity: Role of Education and Income

**DOI:** 10.3390/ijerph192315931

**Published:** 2022-11-29

**Authors:** Zhiwei Dong, Liping Wu, Yang Chen, Oleksii Lyulyov, Tetyana Pimonenko

**Affiliations:** 1 School of Sports Science, Fujian Normal University, Fuzhou 350117, China; 2 College of Economics and Management, Jiangxi Agricultural University, Nanchang 310045, China; 3 School of Economics, Fujian Normal University, Fuzhou 350117, China; 4 Department of Management, Faculty of Applied Sciences, WSB University, 41-300 Dabrowa Gornicza, Poland; 5 Department of Marketing, Sumy State University, 40007 Sumy, Ukraine

**Keywords:** income, income inequality, education, obesity, health outcome

## Abstract

Based on the sixth round of the 2018 Chinese Household Income Project family income survey (CHIP) data, this study made use of the OLS estimation and transfer matrix method to measure and test the problem of obesity intergenerational transmission, analyze whether there is obesity intergenerational transmission as well as between urban and rural areas, gender, and the parental education level and income level on the suppression of the obesity intergenerational transmission effect. The empirical results draw the following main conclusions: obesity intergenerational transmission in Chinese families, the degree of parental obesity has a significant positive impact on the degree of offspring obesity; the higher the degree of parental obesity, the more it can promote the degree of obesity in the offspring. Moreover, the degree of obesity intergenerational transmission is heterogeneous in urban and rural areas and gender. At the same time, the degree of rural obesity intergenerational transmission is higher than that of urban areas, and the degree of male obesity intergenerational transmission is higher than that of women.

## 1. Introduction

With rapid economic development, people are pursuing high-quality living standards, from meeting material quantity to improving the quality of life. In this regard, the word “health” has thus become one of the popular social words frequently appearing, and people have become deeply concerned about and discussed it in recent years. According to the World Health Organization’s definition of health, health is a state of physical and mental perfection and good adaptability, and not just a state of the absence of disease and weakness. This is what people refer to as physical and mental health. That is to say, a person is completely healthy in his physical health, mental health, good social adaptation, and moral health, and he is a completely healthy person. Obesity is the focus of modern people, which has the potential harm to human physical health, mental health, social adaptation, and other aspects. Obesity refers to a certain degree of obvious overweight and fat layers being too thick, and is caused by high body fat, especially excessive triglyceride accumulation. Due to excessive food intake or changes in body metabolism, excessive body fat accumulation causes excessive weight growth and in human pathology, causes physiological changes that may also be latent for some time [1]. The following formula used as the international assessment criteria for obesity: obesity degree = (actual weight-standard weight) standard weight ± 100%. More than 2 billion people are currently overweight or obese worldwide, accounting for a third of the total population [2]. According to a 2013 report released by the Lancet, more than 28% of adult men and 27% of adult women are “overweight” or “obese” [3]. The recent epidemiological survey data showed that the obesity rate for men in China reached 17.10% and the rate for women reached 13.37% [4]; it also showed a clear upward trend. From a medical point of view, obesity is a disease, and excessive body fat accumulation has an adverse impact on health and is a risk factor for inducing hypertension, type 2 diabetes, cardiovascular and cerebrovascular diseases, and a variety of cancers. To some extent, it will reduce life expectancy and bring a significant medical and economic burden to society, of which the World Health Organization lists as one of the top 10 risk factors for the burden of disease.

Based on the global prevalence of obesity and the harm to human health, how to suppress the risk of obesity diseases is worth further study. Therefore, this paper analyzes the mechanism of inhibiting obesity intergenerational transmission from the two aspects of education and income and makes use of OLS to estimate the validity of education and income to inhibit obesity intergenerational transmission, which is of great significance for personal health promotion and China’s health strategy, and also provides a certain basis for the precise health promotion policies in China.

### 1.1. Literature Review

Galton (Francis Galton) first proposed the concept of intergenerational transmission. After this concept was put forward, many scholars have conducted a lot of research on the existence of the intergenerational transmission of income, occupation, education flow, and health, and have achieved fruitful results. In recent years, many scholars have begun to pay attention to the intergenerational transmission of obesity. These studies found that both innate endowment and acquired environment play their own important roles in the intergenerational transmission of obesity. In terms of innate endowment, many scholars believe that parental obesity has a strong positive relationship with offspring obesity. For example, ref. [5] studied the effects of parental health on offspring health inequalities and found that parental obesity significantly increased the probability of offspring obesity, an effect particularly evident in boys. The study carried out by [6] found that for each 1 unit increase in parental BMIZ, the parental BMIZ score will increase by about 20%. The study [7] estimated the degree of father and mother transmission to offspring health, respectively, and found that for each 1 unit increase of father and mother, the BMIZ score of offspring was increased by 0.205 and 0.164 units, respectively. There was no significant difference between the father and mother in health transmission. The past study [8] found a BMI intergenerational elasticity of 0.2 in the sample of biological children but found no significant intergenerational association in the sample of adopted children.

At the level of acquired environment, many scholars believe that the acquired environment, such as education, economic income, and living habits, plays an important influence in the intergenerational transmission of obesity. Some scholars believe education is considered the most important health-influencing factor [9]. This is because education endows individuals with a healthier risk perception and stronger “self-control beliefs”, to avoid and resist health risk behaviors such as smoking and alcohol abuse and to adhere to a healthy lifestyle for a long time [10,11]. There are also studies suggesting that fathers with higher education increase the probability that the offspring have good physical health. With the improvement of paternal education levels, the BMIZ scores have decreased in the offspring [12]. The reason for this may be that education can influence the perception of health and related health behaviors [13]. The higher the paternal education level, the more inclined they are to have good living habits and health care awareness, pay more attention to the cultivation of good health habits of their children, and help them defend themselves against bad health behaviors, such as active exercise and control of tobacco and alcohol intake [14]. Obesity incidence rates are gradually increasing and are high worldwide because of unhealthy eating habits and an inactivity lifestyle [15].

On the other hand, the higher the health cognition level of fathers, the richer their health knowledge and the better their understanding of health publicity materials and medicinal instructions to improve the health production efficiency of themselves and their offspring [16]. Studies have also found that people with higher economic incomes tend to have better physical function [17] because income directly affects the parental health investment in the offspring. Increased parental health investment in the offspring can improve the health level of the offspring by paying for health, providing balanced nutrition, and improving genetics [18]. Healthy behaviors can also be promoted by paying for healthy, balanced meals, and other costs [19]. Additionally, it can reduce the living pressures due to low income and poverty [20]. Alternatively, the differential effects of education on health among different groups may be related to income, because income is an important mediator linking education and health [21]. Some studies believe that in the intergenerational transmission of obesity, innate endowment and acquired environment may interact [22]. The performance of genetic factors depends on the acquired environment. If the acquired environment is more favorable, then the negative factors in the parent gene will not appear in the offspring, otherwise it is more obvious.

Some scholars can analyze the problem of obesity from the perspective of gender and urban and rural residents. Kong Guoshu and Qi Yaqiang [23] confirm that the obesity problem is concentrated in urban men and rural women. The relationship between income and obesity has obvious nonlinear characteristics, including middle income people and urban and rural areas and income; with a higher income risk than lower income groups, there was no significant correlation between high income and obesity in women and urban residents. Some scholars [24] have found that the rates of the overweight and obese population are higher in the urban and economically developed areas than the rural and economically backward areas, and that the coastal economic development level in the proportion of the obese population is close to the average level of developed countries, while the proportion of the obese population in inland rural areas is much lower. Scholars [7] have justified that the intergenerational transmission of rural obesity is higher than that in cities, possibly due to the large differences in health conditions, infrastructure and living conditions between urban and rural areas, all of which will affect the intergenerational transmission of obesity. The study by [25] confirmed this view from the analysis of health intergenerational transmission between urban and rural household registration differences.

According to the review and combing of the existing literature, it is known that from the research perspective, most existing studies use obesity as the basic measure of healthy intergenerational transmission, and the dynamics of intergenerational transmission of obesity itself are insufficient. In terms of research content, the existing studies mainly focus on the relationship and mechanism of obesity intergenerational transmission and innate inheritance, and few studies explore the extent and mechanism of acquired environmental factors to inhibit obesity intergenerational transmission. In view of this, this paper is based on the complexity of obesity congenital genetic and postpartum obesity innate genetic factors, which is not discussed, mainly from the perspective of the acquired environment, and explore two aspects of education and income to suppress the obesity intergenerational transmission mechanism, and use OLS to estimate education and income to suppress the validity of obesity intergenerational transmission.

#### 1.1.1. The Mechanism of Educational Inhibition of the Intergenerational Transmission of Obesity

Education will play an increasingly important role in the Outline of the Healthy China 2030 Plan, the Opinions, and the Healthy China Initiative (2019–2030) [25]. Higher educated parents increase the probability of good physical health [26], and parental health cognition, health behavior, health knowledge, and so on have non-negligible effects on intergenerational health transmission [27]. It can be seen that the parental education level should play a positive role in suppressing obesity intergenerational transmission. The higher the education level, the better the effect of inhibiting obesity intergenerational transmission. The mechanism of action is mainly summarized in the following three aspects: First, usually the higher the level of education, the relatively higher level of healthy cognition. With the development of the economy, food has become richer and higher in fat and sugar, including foods such as chocolate, KFC, and cream, favored by children and teenagers, along with electronic products generally introduced to families. Children and teenagers cannot resist the temptation of electronic products, leading to mobile phone addiction and a lack of outdoor activities, and these factors can then lead to children’s obesity, thus having a negative effect. Good parental health awareness can have a clearer understanding of the importance of health in the life cycle, and the harm of obesity in the life cycle. Therefore, a father with a high level of education not only requires himself f to form good living habits and health awareness [28], he pays more attention to the cultivation of his offspring’s health concepts and health behavior management, resists bad habits in life, and helps his children choose a more healthy diet and establish the consciousness of active participation in physical exercise, and thus can prevent offspring obesity. Second, well-educated parents have a higher health knowledge. The richer the health knowledge, the better understanding of the formation principle of obesity, what factors will lead to the occurrence of obesity, and how these factors will lead to physical obesity, so as to better health education and life management of the offspring, to avoid the occurrence of obesity. In addition, reasonable nutrition and frequent exercise are important means to inhibit physical obesity effectively. To make good use of these two means, it is necessary to have professional knowledge in the field of nutrition and physical education, and it is relatively difficult for the groups with low education levels to master. The high education level group has a strong learning ability, logical thinking ability, knowledge and information collection ability, and modern technology and equipment application ability, which can be easier within the system to master the professional knowledge of the two fields, for the offspring’s healthy diet, scientific sports fitness to provide guidance and help, and control the offspring’s body mass index control within a reasonable range. Finally, well-educated parents have better health behaviors themselves.

The family ecosystem model believes that there are similar living habits and norms in the family system, with a homegroup effect among family members in the same ecosystem. The parental health behavior will bring a demonstration effect on the offspring [29]. Generations learn through intergenerational imitation. Bad behaviors such as parental smoking, drinking, snacks, and high fat foods are also inherited or continued by their offspring [30]. Some studies believe that parental smoking and drinking were highly positively associated with offspring smoking and drinking [31,32]. The same is believed for fat intake and dietary behaviors such as eating snacks [33,34]. These bad behaviors are the main factors of physical obesity. Therefore, in choosing healthy behaviors, parents should consciously avoid bad health behaviors and inhibit the pathway of bad transmission of obesity intergenerational transmission. However, well-educated parents have a strong sense of health. They can consciously resist bad health behaviors in life and maintain their own good health state, thus indirectly affecting the health behavior and lifestyle of the offspring [35], which is conducive to inhibiting the intergenerational transmission of obesity.

#### 1.1.2. Mechanism of Income Inhibition of the Intergenerational Transmission of Obesity

A healthy diet is an important means to restrain physical obesity. In the period of the extensive economic development mode, Chinese residents mainly need physical labor. They need a lot of energy to support the consumption of physical labor, but due to their low income, they can only choose affordable rice, flour, and other high-sugar and fatty foods. With the rapid development of China’s economy and the transformation and upgrading of economic structure, the production form has changed from physical labor to mental labor, and physical energy consumption has been relatively reduced. The original diet structure of residents will lead to excess energy and substances in the body, and a large accumulation of fat cells, resulting in physical obesity [36]. Therefore, the dietary structure of residents needs to be changed, requiring a balanced intake of various nutrients, paying more attention to the quality of food rather than quantity, choosing healthier food, and avoiding excessive intake of high fat, high cholesterol, and high calorie foods, thus avoiding the excess for the offspring, which is where the hidden danger lies for the offspring. Adjusting diet structure will inevitably bring about an increase in economic cost, which needs a certain economic strength as support. Therefore, the increase in family economic income can effectively guarantee the choice of a healthy family diet structure, meet the nutritional needs of the body growth and development of the offspring, and shape the physical physique of the offspring. Exercise is a good doctor.

Exercise not only promotes health and disease prevention but also is an important means of weight regulation [37]. With China’s innovation-driven, rapid change of science and technology to bring more convenience to our daily life, children have convenient transportation tools, buildings have elevators, which also leads to the lack of physical activity of children, coupled with sufficient and abundant edible substances, and may lead to the imbalance of human energy metabolism caused by body obesity. This requires outdoor physical activity added to the intervention to achieve a balance of the body’s energy metabolism. Sports activities are particularly different from other general social activities; they involve rapid movement, instant jumping, physical contact, and contact between people and field equipment. There is no doubt that the sports process contains many risk factors [38]. Therefore, parents need to create a safe sports activity environment for their children, such as appropriate clothing, safe sports equipment, and scientific training and guidance services, which requires a high economic investment. The improvement of family income, for children to participate in sports equipment and training services to buy financial guarantee, let the offspring in a comfortable environment be with a professional for the guidance of sports fitness activities, who can stimulate the interest of the children in sports fitness, normalized fitness habits, and this can effectively control the body mass index in a reasonable range. Superior medical care is also an important factor in blocking obesity. Higher family income can provide better health care services for their children [39]. Through regular physical health monitoring and evaluation, scientific exercise prescriptions and nutritional formulas are formulated for the offspring, to avoid the body obesity caused by the excess energy caused by eating more and doing less. Through regular physical health medical examinations, a poor physical condition in the offspring can be timely and accurately found, and scientific medical intervention can be conducted early to block the deterioration of the condition and avoid secondary obesity. In sum, the increase in parental economic income, will lead to an increased investment in the health of the offspring [40], by way of paying health care services, improving diet and sports fitness investment ability, which will be stronger, so that the offspring receives better health services, balanced nutrition, and diversified sports fitness services; hence, this results in better improvements in the health level of the offspring, effectively suppressing intergenerational obesity transmission.

## 2. Materials and Methods

### 2.1. Data Source

The data of this study were obtained from the sixth round of the China Household Income Project (CHIP) in 2018, which is regarded as the most comprehensive and rigorous public database on micro-incomes in China. The China family income survey is organized by the National Bureau of Statistics, the Institute of Economics of the Chinese Academy of Social Sciences, and other units. It aims to measure and estimate the income distribution of urban and rural residents in China and generates samples through secondary samples from a large sample of urban survey teams and agricultural survey teams of the National Bureau of Statistics. Six rounds of surveys have been conducted: 1988, 1995, 2002, 2007, 2013, and 2018. By 2018, the CHIP covered information on personal physical characteristics, self-rated health, education, work, and income of 160,000 residents in 31 provinces, which is very suitable for studying issues related to the intergenerational transmission of obesity. Considering that in the vast majority of families, fathers occupy a dominant position in family income and decision making, the relevant information of fathers was selected as the parental sample, and the children’s data were selected to match, data were then combined, duplicate observations were processed for multi-child families, and samples such as missing and inapplicable key variables were eliminated, and finally the overall effective sample size was 3055 pairs.

### 2.2. Variable Selection

In this paper, the dependent variable was the obesity status of the offspring, with BMI and values as a measure. BMI (body mass index), or BMI index, also known as body mass index, is an important measure of obesity and standard weight. The criteria set by the World Health Organization (WHO) are divided into seven grades: thin (<18.5), normal (18.5~24.9), overweight (≥25), obese (25.0~29.9), obese (30.0~34.9), severe obesity (35.0~39.9), and extremely severe obesity (≥40.0). The independent variable is the obesity status of its parent. At the same time, the income level and education level of the parent generation were selected as the control variables.

### 2.3. Model Setting

On the measure of the intergenerational transmission of obesity, this study draws the concept of intergenerational income elasticity (IGE) in economics and proposes intergenerational obesity elasticity. Intergenerational obesity elasticity is regarded as the main measure of obesity intergenerational transmission, mainly by examining the correlation between the degree of offspring obesity and the degree of paternal obesity, to determine how far the level of offspring obesity is determined by the level of paternal obesity. The higher the correlation between the offspring obesity levels and the paternal obesity levels means that the greater the extent the offspring obesity level is determined by the paternal obesity levels. This also means that the paternal obesity gap is transmitted with intergenerational obesity, affecting and even determining the obesity gap between offspring. Therefore, the use of intergenerational obesity elasticity can measure the degree of intergenerational obesity transmission in a relative sense.

(1) Benchmark Model of Obesity in Intergenerational Transmission

Based on the concept of the intergenerational transmission of obesity, this paper uses the linear regression model to investigate the intergenerational obesity transmission of Chinese residents, which is also commonly used research to measure the elasticity of the intergenerational obesity. The measurement model is as follows:

(1) $$BMI_{ci}=\alpha +\beta BMI_{fi}+e\;\;\;\;$$

where, $BMI_{ci}$—BMI of the i-th family; $BMI_{fi}$ represents the BMI of the parent in the i-th family; α represents the constant term; e represents the residual term, β indicates the intergenerational obesity elasticity, which is the main measure of the intergenerational transmission degree of obesity, that is, the larger β means that the greater the influence of offspring obesity by paternal obesity, the stronger the intergenerational transmission of obesity; the smaller β means that the smaller the offspring obesity is affected by paternal obesity. Among them, the main method to obtain intergenerational obesity elasticity is by using least squares (OLS), in *Cov*(*ln*$BMI_{fi}$, Under the assumption of e) = 0, where the OLS estimate is as follows:

(2) $$\beta =\frac{Cov\left(InBMI_{fi},InBMI_{ci}\right)}{Var\left(InBMI_{fi}\right)}$$

(2) Intergenerational Obesity Transfer Matrix

The intergenerational obesity transfer matrix is able to describe the position of the offspring of different obese groups in their peers, intuitively describing the degree of the intergenerational transmission of obesity. Intergenerational obesity transfer matrix $P_{\mathrm{ij}}$ = [$P_{\mathrm{ij}}$_j_X and Y in (X, Y)] are the obesity distributions of the parent and the offspring, respectively, and X- -Y indicates the obesity distribution to transition from X of the parent to Y of the offspring, and each element P in the matrix_ij._ Parent represents the probability of offspring in the i-th group and offspring in the j-th group.

The intergenerational obesity transfer matrix was calculated as follows. First, the paternal population and offspring population samples were divided into five BMI index levels according to their obesity levels, marking the level of paternal obesity and offspring obesity in each family sample. Then, using the parent obesity as the benchmark, the calculation results of each obesity level were calculated in the matrix form to obtain the intergenerational obesity transfer matrix, The specific form is as follows:

(3) $$P_{\mathrm{ij}}=\left[\begin{array}{cccc} P_{11} & P_{12} & P_{13} & P_{14}\\ P_{22} & P_{22} & P_{23} & P_{24}\\ P_{31} & P_{32} & P_{33} & P_{34}\\ P_{41} & P_{42} & P_{43} & P_{44} \end{array}\right]$$

where the element $P_{\mathrm{ij}}$ represents the parent in the i-th BMI group, whose child income is in the ratio of the j-th BMI group, and the sum of each row of data is equal to 1.

(3) Correlation model of obesity intergenerational transmission inhibition pathway

Correlation models of obesity intergenerational paths look at identifying the role of different classes of intermediate variables in intergenerational transmission. It is mainly to place the corresponding intermediate variables in the simple regression to construct the conditional obesity elasticity, and to obtain the importance of the conditional obesity elasticity by examining the importance of the corresponding intermediate variables on the intergenerational transmission of obesity. The specific model is as follows:

(4) $$BMI_{ci}=\alpha^{*}+\beta_{1}^{*}BMI_{fi}+r_{j}X_{j}+e_{i}^{*}$$

where, $\beta_{1}^{*}$ is the conditional intergenerational obesity elasticity; *X* is the control variable (*j* = 1, 2), $X_{1}$, $X_{2}$. The proxy variable indicates education (EDUF) and income (INCF), and $r_{j}$ is the influence of each proxy variable on offspring obesity.

The ratio of changes in conditional intergenerational resilience relative to intergenerational resilience calculated by analyzing each proxy variable $\left[\frac{\beta^{*}-\beta }{\beta }\;\right]$ examine the influence of various variables on the correlation of obesity between father and son, and thus verify whether the paths of obesity intergenerational transmission are effective.

## 3. Results

### 3.1. Descriptive Analysis of the Main Variables

According to the statistical analysis of each variable, the lowest value of BMI in the offspring was 15.241, the highest value was 28.354, and the mean value was 20.514. The lowest parent BMI value was 15.242, the highest value was 28.345, and the mean value was 23.255. In terms of the level of parental education, the subjects differed greatly, with the highest years of education being 22 years, the lowest years being only 5 years, and an average years of education being 9.268 years. In terms of the economic income of the father generation, the differences between the main subjects were also relatively large. The highest annual income was 1 million yuan, the lowest annual income was 0, and the average annual income was 43.929. Household type and gender were binary variables, assigned 1 for city and 0 for rural areas; 1 for men and 0 for women. See also those listed in (Table 1) for details.

### 3.2. Analysis of Obesity Intergenerational Transfer Rank Transfer Matrix

According to the BMI index standard formulated by the World Health Organization (WHO), this study selected four grades of thin, normal, overweight, and obesity to provide evidence of analysis for the intergenerational transmission of obesity in China. As shown in Table 2: among the 186 people whose parents’ BMI index was “thin”, only two had children whose BMI index was “overweight” and “obese”, accounting for 1.08%, indicating that parents with “thin” BMI had a lower probability of intergenerational transmission of obesity; Among the 2390 parents with a BMI index rating of “normal”, 87 and 24 children had a BMI index of “overweight” and “obese”, respectively, accounting for 3.64% and 1%, respectively, indicating that the probability of intergenerational transmission of obesity in parents with “normal” BMI is relatively low. Among the 446 people whose parents’ BMI index was “overweight”, 42 and 12, whose children’s BMI index was “overweight” and “obese”, respectively, accounted for 9.42% and 2.69%, respectively, indicating that the probability of intergenerational transmission of obesity in parents with “overweight” BMI was higher than that of parents with “thin” and “normal” BMI. Among the 33 people whose parents had a BMI index rating of “obese”, three and six children had a BMI index of “overweight” and “obese”, accounting for 9.09% and 18.18%, respectively, indicating that parents with “obese” BMI had a higher probability of intergenerational transmission of obesity than parents with BMI “thin”, “normal”, and “overweight”. In summary, when the BMI index of the parent generation is in the “thin” and “normal” levels, the probability of upward mobility of the BMI index level of the child is small. When the parent BMI index is in the “overweight” and “obese” levels, the probability of upward mobility of the child’s BMI index level is greater.

### 3.3. Benchmark Regression Outcome Analysis of Intergenerational Obesity Transmission

The results of full-sample regression, as shown in Table 3, *p* = 0.000, indicate that offspring obesity and paternal obesity were significantly correlated, indicating that there was intergenerational transmission of obesity in China. The intergenerational transmission coefficient of obesity was 0.328, indicating that paternal obesity had a significant positive effect on offspring obesity, that is, the higher the degree of parental obesity, the higher the degree of offspring obesity. The intergenerational transmission coefficient of obesity was 0.328, indicating that for every 1 increase in the BMI coefficient of the parent, the BMI coefficient of the offspring increased by 0.328.

From the perspective of urban–rural differences, as shown in Table 3, the intergenerational transmission coefficient of rural obesity was 0.337, *p* = 0.000, indicating that offspring obesity and paternal obesity were significantly correlated, indicating that there was intergenerational transmission of obesity in rural China, and paternal obesity had a significant positive impact on offspring obesity. The intergenerational transmission coefficient of urban obesity was 0.318, *p* = 0.000, indicating that offspring obesity and paternal obesity were significantly correlated, indicating that there was intergenerational transmission of obesity in Chinese cities, and paternal obesity had a significant positive impact on offspring obesity. At the same time, it also shows that there are differences in the intergenerational transmission of obesity between urban and rural areas, and the degree of intergenerational transmission of obesity in rural areas is higher than that in cities.

From the perspective of sex differences, as shown in Table 3, the intergenerational transmission coefficient of obesity between father and son was 0.375, *p* = 0.000, indicating that son obesity was significantly related to paternal obesity, indicating that there was intergenerational transmission of obesity between father and son, and paternal obesity had a significant positive impact on son obesity. The intergenerational transmission coefficient of obesity between father and daughter was 0.270, *p* = 0.000, indicating that daughter obesity and paternal obesity were significantly correlated, indicating that there was intergenerational transmission of obesity between father and daughter, and paternal obesity had a significant positive effect on daughter obesity. At the same time, it also shows that there are differences in the intergenerational transmission of obesity between men and women, and the degree of intergenerational transmission of obesity in sons is higher than that of daughters.

### 3.4. Results of the Inhibition Pathway Test for Obesity with Intergenerational Transmission

According to Table 4 regression results, after controlling for the parental years of education, the benchmark regression coefficient decreased from 0.328 to 0.326, that is, for every 1 increase in the parental BMI coefficient to 0.328 to 0.326. This indicates that education has a negative effect on intergenerational obesity transmission, that is, the higher the level of education, the lower the degree of obesity intergenerational transmission, indicating that improving education has the effect of inhibiting obesity intergenerational transmission. The main reason is that the higher the parental level of education, the higher the health cognition level, the richer the health knowledge, and the more standardized the health behaviors. The improvement of paternal health cognition can mean a more comprehensive understanding of the importance of health in the life cycle process, stimulate the motivation of self to pursue health, and consciously regulate health behavior [8]. At the same time, these health cognition and health behaviors of the fathers will also be applied to the education and management of the offspring, to improve the health cognition and regulate the health behavior, and to avoid the risk of physical obesity. In addition, the parent education level is higher, their learning ability, information literacy ability, and knowledge application ability is relatively high, they can master a relatively rich health knowledge, and can be closely linked to promote health practice; they can therefore combine the offspring health characteristics, age characteristics, gender characteristics, targeted to offspring health promotion, and be more efficient at achieving health goals.

In addition, after controlling for the economic income level of the parent, the benchmark regression coefficient of obesity intergenerational transmission decreased from 0.328 to 0.312, that is, for every 1 increase of the parent BMI coefficient, the added value of the offspring BMI coefficient decreased from 0.328 to 0.312 (see Table 4). This shows that the economic income level has a negative effect on the intergenerational obesity transmission, that is, the higher the economic income level, the lower the obesity intergenerational transmission degree, indicating that increasing the household economic income level has the effect of inhibiting the obesity intergenerational transmission. Its inhibition mechanism is that with the increase in parental income, the health of the offspring investment will increase, including paying for health care services and improvement in diet and sports fitness investment ability being stronger [41], so that the offspring get better health care services, balanced nutrition and diversified sports fitness services, better promoting the health level of the offspring, and effectively suppress intergenerational obesity.

## 4. Discussion

This paper focuses on the intergenerational transmission of obesity in Chinese residents, paternal obesity on offspring obesity, and the greatest impact on fat, further measuring education and income on the intergenerational transmission of obesity in Chinese residents [42]. The results show that there are different relationship patterns between genders and between urban and rural areas. In order to better suppress obesity in Chinese residents, education level and family income level were used. After the parental years of education, the benchmark regression coefficient decreased from 0.328 to 0.326, and the increase in parental BMI coefficient decreased from 0.328 to 0.326. The regression coefficient of the economic income level of the parent decreased from 0.338 to 0.312, that is, for every 1 increase of the parent BMI coefficient, the added value of the offspring BMI coefficient decreased to 0.312 [43]. This shows that the economic income level has a negative effect on the intergenerational obesity transmission, that is, the higher the economic income level, the lower the obesity intergenerational transmission degree, indicating that increasing the household economic income level has the effect of inhibiting the obesity intergenerational transmission [44].

The degree of obesity intergenerational transmission varies between urban and rural areas, with higher intergenerational transmission in rural areas than in urban areas. This result may be due to the large differences in medical and health conditions, and infrastructure and living environment between urban and rural China, all of which can affect the intergenerational transmission of obesity. For rural residents, the restriction of medical infrastructure makes rural residents more likely to fall into the poor transmission of intergenerational obesity. Urban residents themselves have a high health level, and have innate advantages in the utilization of medical resources. Therefore, there is little room for urban residents to improve the intergenerational transmission of obesity. On the other hand, due to the low income in rural areas, low-income families constrained by income invest less in the health of their offspring, and the paternal level of education of rural residents is relatively low, which will ignore the health education of their children to some extent, and it is more likely to lead to the intergenerational transmission of adverse obesity. This also shows that the degree of obesity intergenerational transmission is related to the income gap and the education level. Improving the economic development level of rural areas, increasing the per capita income of residents, improving the medical and health conditions, and the popularization of high education are effective means to curb the intergenerational transmission of obesity.

There are gender differences in the degree of obesity intergenerational transmission, with a higher degree of obesity intergenerational transmission in men than in women. Specifically, boys’ BMI values tend to be higher, which may be more pronounced in traditional Chinese family parenting culture, especially in rural societies or intergenerational parenting families. In addition, in Chinese social culture, women hold the concept of thin beauty as a dominant position in society. Therefore, women will actively take some behaviors to control their weight and maintain a slim and symmetrical body shape. On the other hand, women are in a weak position in the labor market and are vulnerable to discrimination in the labor market. In order to avoid the negative effects of obesity, women pay more attention to their own health investment and healthy lifestyle, and increase exercise time to control the obesity index.

## 5. Conclusions

Based on the sixth round of the 2018 Chinese family income survey (CHIP), using the variable method tool of empirical test of Chinese residents’ obesity intergenerational transmission problems, the following main conclusions can be drawn:

1. Improving the parental education level can effectively inhibit the intergenerational transmission of obesity. First, with the improvement of parental education level, parental health cognition level also increased simultaneously. The improvement of parental health cognition can have a more comprehensive understanding of the harm of obesity on physical function and the negative impact on career development, social communication, psychology, and other aspects. Promoting parental scientific intervention of the offspring, such as healthy diet, regular physical activity, and behavior, standardized living habits, and the input of healthy ideas, can effectively avoid the occurrence of offspring obesity. Second, the improvement of parental education level, its learning ability, information literacy ability, and knowledge application ability, knowledge is relatively high, relatively rich, and can closely be linked to health promotion practice, and combined with offspring health characteristics of age and gender, and targeted offspring health promotion, it is more efficient in achieving health goals and avoid the risk of offspring obesity. Finally, the improvement of paternal health cognition can have a more comprehensive understanding of the importance of health in the life cycle process, stimulate the motivation of self to pursue health, and consciously regulate health behavior [8]. In the family system, there is a family group effect among family members. Parent health behavior will bring a demonstration effect to the offspring. Through intergenerational imitation learning, parental health behavior will also be inherited or continued by the offspring [45]. In addition, these parental health cognition and health behaviors will also be applied to the education and management of the offspring, to improve their health cognition and regulate their health behavior, and to avoid the risk of physical obesity.

2. Increasing the parental income level can effectively inhibit the intergenerational transmission of obesity. At present, healthy diet, regular sports, and perfect health care services are the common ways to control and prevent overweight and obesity [23]. These ways are not easy to obtain, as they require paying high costs and certain economic strength as support. As parent economic income increases, the health of offspring investment will increase [40], payment for health services will increase, and the improvement in diet and sports fitness investment ability is stronger, so as to obtain better medical and health services, nutrition balance, and diversified sports fitness services, and will better improve the health level of the offspring, and effectively suppress intergenerational obesity.

Despite the valuable results of the investigation, the paper has a few limitations. Thus, it would be better to extend the sample for analysis (by number and geography) which allow obtaining more reliable conclusion.

## Figures and Tables

**Table 1:** Descriptive statistics of main variables (N = 3055).

Variable	Mean	Standard Deviation	Least Value	Crest Value
Father Generation				
BMI	23.01	2.66	15.65	36.73
Education level	11.36	2.72	0	22
annual earnings	5.966	0.94	0.06	100
Household type (city = 1)	-	-	0	1
Sex (male = 1)	-	-	0	1
Offspring				
BMI	20.98	2.69	15.237	37.253
Household type (city = 1)	-	-	0	1
Sex (male = 1)	-	-	0	1

Source: Authors’ own elaboration.

**Table 2:** Grade transfer matrix of obesity intergenerational transmission (N = 3055).

Variable		Secondary-Generation BMI Index			
		Thinnish (<18.5)	Normal (18.5–24.9)	Overload (≥25)	Fat (≥30)
Parent-generation BMI index	Thinnish (N = 186)	23.12% (N = 43)	74.72% (N = 139)	1.08% (N = 2)	1.08% (N = 2)
	Normal (N = 2390)	10.67% (N = 255)	84.69% (N = 2024)	3.64% (N = 87)	1.00% (N = 24)
	Overload (N = 446)	6.50% (N = 29)	81.39% (N = 363)	9.42% (N = 42)	2.69% (N = 12)
	Fat (N = 33)	9.09% (N = 3)	63.64% (N = 21)	9.09% (N = 3)	18.18% (N = 6)

Source: Authors’ own elaboration.

**Table 3:** Baseline regression results for intergenerational obesity transmission.

Variable	Full Sample	Census Register		Sex	
		Town	Rural Area	Man	Woman
BMI	0.328 ***	0.318 ***	0.337 ***	0.375 ***	0.270 ***
*p*-value	0.000	0.000	0.000	0.000	0.000
R^2^	0.076	0.075	0.077	0.058	0.089
F value	250.747	120.36	130.5	95.13	149.36
observed value	3055	1485	1570	1540	1515

Note: *** means *p* < 0.001; Source: own elaboration

**Table 4:** Results of the inhibition pathway for intergenerational transmission in obesity.

Variable	Model 1	Model 2	Model 3
BMIF	0.328 ***	0.312 ***	0.326 ***
*p* price	0.000	0.000	0.000
controlled variable	-	EDUF	INCF
R^2^	0.097	0.077	0.083
F value	250.747	127.78	137.96

Note: *** means *p* < 0.001, EDUF—education; INCF—income. Source: Authors’ own elaboration.

## Data Availability

Not applicable.
